# The Importance of Advancing Research on Aging and Driving

**DOI:** 10.3390/geriatrics6010007

**Published:** 2021-01-14

**Authors:** Samantha A. Murphy, Ganesh M. Babulal, Catherine M. Roe

**Affiliations:** Knight Alzheimer Disease Research Center, Department of Neurology, Washington University School of Medicine, St. Louis, MO 63110, USA; babulalg@wustl.edu (G.M.B.); cathyr@wustl.edu (C.M.R.)

Between 2009 and 2018, the number of older adults (i.e., 65 years and above) in the United States increased by 32%, and this number will continue to grow in the coming decades as people live longer than ever before [1]. Approximately 20 older adults are killed and 700 injured daily in motor vehicle collisions, making up 19% of all traffic fatalities across the United States [2]. As driving is a complex task, it requires older adults to utilize a variety of skills, including physical, cognitive, and perceptual abilities. Due to age-related declines in physical health and cognitive ability, driving becomes more difficult for older adults [3]. Some age-related changes, such as reduced reaction time and visual acuity, are risk factors for the deterioration of driving performance and driving decline [4]. Driving performance and crash risk are also known to be affected by numerous medical and neurological conditions that are more prevalent in older age, such as cardiovascular disease, diabetes, Parkinson’s disease, Alzheimer’s disease, and stroke [5,6]. With the number of older adult drivers increasing over the next few decades, the importance of aging and driving research will become more crucial, and a better understanding of driving behavior and risk in older adults is needed. This knowledge can assist geriatric clinicians in helping older adults, including those with neurological diseases, make driving cessation and mobility decisions, and can better inform clinical practice.

This Special Issue of Geriatrics is focused on the important topic of aging and driving, and consists of 11 reviews, case reports, and commentary articles that include a variety of quantitative and qualitative methodologies. As a group, these articles thoughtfully examine aging and driving in the contexts of Alzheimer’s disease, cognitive impairment, environmental risks, planning for driving cessation, and naturalistic driving. The novel information presented in these articles aids in further understanding driving in older adult populations.

The first article in this Special Issue utilized the A/T/N framework to examine driving in preclinical Alzheimer’s disease (AD), the long stage preceding the onset of clinical dementia symptoms. Originally developed by Jack and colleagues [7], the A/T/N framework encompasses seven major AD biomarkers and divides them into three categories (β-amyloid, tau, and neurodegeneration or neuronal injury) based on the pathology that each measure. Roe and colleagues found that the onset of driving difficulties is more highly associated with abnormal levels of both amyloid and tau biomarkers, rather than amyloid alone. Their findings suggest that this framework, designed to guide research among those with memory and thinking difficulties, may also be useful in guiding research involving functional outcomes, such as driving.

In a first-of-its-kind study, Hird et al. combined driving simulations and functional magnetic resonance imaging (fMRI) to investigate brain function in individuals with mild cognitive impairment (MCI) while performing a simulated driving task. These researchers found that individuals with MCI had a greater difficulty with lane maintenance when compared to healthy controls. Individuals with MCI also had reduced connectivity in regions involved in visual attention and processing, cognitive control, and performance monitoring. This suggests that participants with MCI have increased difficulty in performing some tasks important to safe driving.

In addition to those with MCI, all older adults will show a decline in safe driving skills and will eventually need to cease driving if they live long enough. The next article in this issue, by Harmon et al., quantified behaviors and beliefs related to planning for driving cessation among middle-aged driving adults. In their sample composed of a majority of black and female participants in urban Detroit, they found that less than half had planned for a non-driving future. Although there were very low levels of planning behaviors reported, a vast majority stated that they believe planning would help them after driving cessation, especially with the emotional transition (i.e., emotions following not being able to regularly attend normal activities, loss of productivity, and inability to maintain connections). The drivers that did plan for cessation were older, drove less frequently, and expected to continue driving three years less than those who did not plan.

The following article by Knoefel and colleagues discusses a framework for driving assessments, including information as to how naturalistic driving studies can provide increased assistance to other driving assessment methods, such as neuropsychological testing or on-road assessments. Naturalistic driving methods allow for reducing participant burden while collecting important data and may soon aid clinicians in evaluating driving behaviors. As well, they can assist in making driving cessation decisions through clear observation of the participant’s everyday driving behaviors such as driver actions and reactions, driving conditions, and destinations visited.

Naturalistic driving was also the focus of Davis et al. They evaluated the use of an automated data reduction system to determine if it was more effective in identifying situations in which individuals may be at higher risk of driving errors. It was found that those with AD tended to have increased difficulty with the fundamentals of driving, specifically lane maintenance and anticipation of traffic situations and maneuvers, while cognitively healthy older adults made increased errors due to multi-tasking or being distracted by technology within the vehicle. Davis and colleagues found that the automated data reduction system achieved the highest sensitivity and specificity in predicting an AD diagnosis, suggesting that this analysis could prove more informative in clinical applications than the composite ratings currently used.

The following two articles by Huisingh et al. were prospective studies that utilized a population-based sample of 2000 licensed adults to investigate forms of cognition, assessed by mental state screeners, as risk factors for a vehicle collision. The first article examined if cognitive impairment or decline, when measured by a brief mental status screener, is associated with crash risk. It was found that crash involvement was higher for those with cognitive impairment. Huisingh and colleagues also found that drivers who experienced a cognitive decline were 1.64 times more likely to have a motor vehicle accident in which they were at fault. The second article assessed the relationship between areas of cognitive functioning on the Mini-Mental State Examination and the risk of motor vehicle crash involvement. In this article, Huisingh and colleagues reported that those with spatial orientation difficulties were six times more likely to be involved in a motor vehicle crash, suggesting that spatial orientation impairment may be able to help clinicians identify older adult drivers that are at risk for motor vehicle collisions.

Motor vehicle collisions were also the focus of the next article by Payyanadan et al. They performed a qualitative review of the literature on differences in motor vehicle collision outcomes, and how these may be attributed to challenges older drivers are facing based on residence location. Focus groups were utilized to interview older adult drivers and discuss challenges focused on setting and location. It was found that older adults often use avoidant behavior and route selection to address these challenges. It is possible that innovative driver support system technology features, such as pre-trip planning and post-drive feedback, could be tailored to address these challenges in older drivers that live in urban, suburban, and rural areas.

The following systematic review by Babulal et al. discussed the issue of the US population living and driving longer than ever before, as well as the population of older adults becoming more racially and ethnically diverse over the next three decades. This systematic review included 18 studies on driving and racial or ethnic differences among older adults. Across the articles reviewed, Babulal and colleagues found that racial and ethnic minorities were at greater risk for reduced driving, driving cessation, and mobility restrictions. The authors suggest that more prospective observational driving studies should be inclusive of ethnic and racially diverse populations.

The next two articles revolved around the importance of resources and technology. The first article by Dickerson et al. was in the form of a commentary discussing potential options and strategies for creating evidence-based fitness to drive decisions. Dickerson and colleagues noted the importance of differentiating between driving skills and capacities, as well as how these are demonstrated while on the road. The authors discussed a framework for more easily viewing and comparing programs and resources for driving, the Spectrum of Driver Services. Finally, Maze and Hunt presented a case report about an older adult man with dementia and his difficulty in learning to utilize a smartphone. This case report highlighted the importance of both caregiver and patient learning, especially when it comes to enhancing safety during daily activities.

To summarize, the rich collection of studies and article types in this Special Issue adds new information and insight to the existing literature on aging and driving. The 11 articles utilized various older adult populations and assessed novel frameworks, as well as predictors and risk factors, for specific driving behaviors using a variety of methodologies. Most importantly, they shed light on areas of research that are lacking, including the involvement of additional racial and ethnic groups in studies and the need to make room for more natural, efficient, and less intrusive driving assessments. We hope you enjoy reading these articles as much as we did.
